# Real-world corticosteroid use in severe pneumonia: a propensity-score-matched study

**DOI:** 10.1186/s13054-021-03840-x

**Published:** 2021-12-16

**Authors:** A. Ceccato, A. Russo, E. Barbeta, P. Oscanoa, G. Tiseo, A. Gabarrus, P. Di Giannatale, S. Nogas, C. Cilloniz, F. Menichetti, M. Ferrer, M. Niederman, M. Falcone, A. Torres

**Affiliations:** 1 Ciber de Enfermedades Respiratorias (Ciberes, CB06/06/0028), Institut d’Investigacions Biomèdiques August Pi I Sunyer (IDIBAPS), University of Barcelona (UB), Barcelona, Spain; 2 Department of Public Health and Infectious Diseases, Policlinico Umberto I, “Sapienza” University of Rome, Rome, Italy; 3 Department of Pneumology, Institut Clinic de Respiratori, Hospital Clinic of Barcelona, Villarroel 170, 08036 Barcelona, Spain; 4 Infectious Diseases Unit, Department of Clinical and Experimental Medicine, University of Pisa, Pisa, Italy; 5 Department of Medical, Oral and Biotechnological Sciences, School of Medicine and Health Sciences, Section of Anesthesia Analgesia, Perioperative and Intensive Care, SS. Annunziata Hospital, Gabriele d’Annunzio University of Chieti-Pescara, Chieti, Italy; 6 Dipartimento Scienze Chirurgiche E Diagnostiche Integrate (DISC), Università Degli Studi Di Genova, Genova, Italy; 7 Division of Pulmonary and Critical Care Medicine, Weill Cornell Medical College, New York Presbyterian/Weill Cornell Medical Center, New York, NY USA

**Keywords:** Community-acquired pneumonia, Corticosteroids, Mortality

## Abstract

**Background:**

Community-acquired pneumonia (CAP) is a leading cause of morbidity and mortality worldwide despite correct antibiotic use. Corticosteroids have long been evaluated as a treatment option, but heterogeneous effects on survival have precluded their widespread implementation. We aimed to evaluate whether corticosteroids might improve clinical outcomes in patients with severe CAP and high inflammatory responses.

**Study design and methods:**

We analyzed two prospective observational cohorts of patients with CAP in Barcelona and Rome who were admitted to intensive care with a high inflammatory response. Propensity score (PS) matching was used to obtain balance among the baseline variables in both groups, and we excluded patients with viral pneumonia or who received hydrocortisone.

**Results:**

Of the 610 patients admitted with severe CAP, 198 (32%) received corticosteroids and 387 had major criteria for severe CAP. All patients had a baseline serum C-reactive protein above 15 mg/dL. Patients who received corticosteroids were more commonly male, had more comorbidities (e.g., cancer or chronic obstructive pulmonary disease), and presented with significantly higher sequential organ failure assessment scores. Eighty-nine patients met major severity criteria (invasive mechanical ventilation and/or septic shock) and were matched per group. Twenty-eight-day mortality was lower among patients receiving corticosteroids (16 patients, 18%) than among those not receiving them (28 patients, 31%; *p* = 0.037). After PS matching, corticosteroid therapy reduced the 28-day mortality risk in patients who met major severity criteria (hazard ratio (HR) 0.53, 95% confidence interval (CI) 0.29–0.98) (*p* = 0.043). In patients who did not meet major severity criteria, no benefits were observed with corticosteroid use (HR 0.88 (95%CI 0.32–2.36).

**Conclusions:**

Corticosteroid treatment may be of benefit for patients with CAP who have septic shock and/or a high inflammatory response and requirement for invasive mechanical ventilation. Corticosteroids appear to have no impact on mortality when these features are not present.

**Supplementary Information:**

The online version contains supplementary material available at 10.1186/s13054-021-03840-x.

## Background

Community-acquired pneumonia (CAP) is a leading cause of mortality and morbidity worldwide despite correct antibiotic use [1, 2]. Severe disease not only manifests frequently and requires intensive care unit (ICU) admission [3, 4], but also presents with the highest mortality [5]. Death in severe CAP occurs due to an overwhelming pulmonary and systemic inflammatory response that causes abnormal gas exchange (i.e., respiratory failure), sepsis, and multiple-organ dysfunction [6]. Although antimicrobials are highly effective at reducing the bacterial burden of pulmonary infection [7], they do not directly modulate the inflammatory response.

Poor clinical outcomes in severe CAP necessitate treatment strategies other than antibiotics. Corticosteroids are a biologically plausible option that inhibit the expression and action of cytokines involved in the inflammatory response associated with pneumonia [8]. However, their use in CAP has yielded heterogeneous survival results in randomized clinical trials, in part because they included patients with and without severe CAP, precluding their routine inclusion in therapeutic strategies [9]. Appropriate selection of patients with severe CAP only, in whom the beneficial effects of corticosteroids outweigh the potential adverse effects, is therefore of major relevance [10, 11]. Indeed, subgroup analyses of patients treated for CAP with corticosteroids have shown survival benefits mainly in those with severe disease. This is plausible because the low mortality in patients without severe CAP makes it less likely that we will observe reduced mortality [12–18]. Given that the rationale for corticosteroid use is to attenuate the inflammatory response, those with the highest inflammatory status should obtain the most benefit. In a randomized clinical trial of patients with severe CAP and high C-reactive protein (CRP) levels, corticosteroid use led to less late radiographic progression of pulmonary opacities [19]. However, this study was underpowered for assessing differences in mortality and other clinical outcomes.

We hypothesize that corticosteroids might improve clinical outcomes in patients with severe CAP and high inflammatory responses by reducing pulmonary and systemic inflammation. Accordingly, we aimed to analyze the impact of corticosteroids on mortality and other outcomes in patients with severe CAP and a high inflammatory response.

## Study design and methods

This was an observational multicenter study in a real-world setting, using data from two prospective cohorts of consecutive patients with CAP admitted to the Hospital Clinic of Barcelona in Spain (January 2004 to December 2019) and the Policlinico Umberto I, “Sapienza” University of Rome in Italy (January 2015 to December 2017). The inclusion criteria were as follows: (a) adults aged ≥ 18 years at diagnosis; (b) CAP confirmed by chest radiograph with consistent clinical manifestations (e.g., fever, cough, sputum production, and pleuritic chest pain); (c) patients admitted to ICU; (d) patients with a high inflammatory response, defined as a CRP > 15 mg/dL at admission, based on the results of a previous study [19], (e) patients with major criteria for severe CAP according to ATS/IDSA criteria at baseline (24–48 h) [4]. The following exclusion criteria were also applied: (a) hospital admission for ≥ 48 h in the preceding 14 days; (b) absence of complete clinical follow-up data for 4–6 weeks; (c) severe immunosuppression, such as post-transplantation, HIV co-infection, or chemotherapy or immunosuppressive drug use (> 20 mg prednisone equivalent per day for ≥ 2 weeks); (d) confirmed viral infection; and (e) hydrocortisone use.

### Ethics statement

The ethics committees of the Hospital Clinic of Barcelona (Register: 5451) and of Policlinico Umberto I (register: 4065) approved the study and its publication. The need for written informed consent was waived because of the non-interventional design. Patient identities remained anonymous throughout.

### Data collection

Details of comorbidities were obtained from medical records. Clinical, laboratory, and radiographic characteristics were recorded on admission (described in detail in Additional file 1: Content). During hospitalization, the following data were recorded: length of stay, need for mechanical ventilation (including whether invasive [IMC] or noninvasive [NIMV]), and 30-day mortality. Severe CAP was defined according to the American Thoracic Society and Infectious Diseases Society of America (ATS/IDSA) guidelines [20]. The Pneumonia Severity Index (PSI) [3] and Sepsis-related Organ Failure Assessment (SOFA) [21] scores were used to stratify cases by severity.

### Definitions

Patients were grouped by their initial treatment into those who did and did not receive corticosteroids (prednisone, methylprednisolone, dexamethasone), which was considered to have been given if the patient received at least 72-h treatment with prednisone or an equivalent at a dosage of ≥ 30 mg/day in the first 48 h of admission. Septic shock was defined according to the ATS/IDSA pneumonia guidelines [4]. Empiric antimicrobial treatment was deemed appropriate when the isolated pathogens had in vitro susceptibility to at least one of the administered antimicrobials or follow ATS/IDSA guidelines [4] recommendation if no pathogen was isolated.

### Outcomes

The main outcome was the 28-day all-cause mortality. The secondary outcome was the length of stay in hospital.

### Statistical analysis

We reported the number and percentage of patients for categorical variables, the median and first and third quartiles for continuous variables with non-normal distributions, and the mean and standard deviation for continuous variables with normal distributions. Categorical variables were compared using Chi-square or Fisher exact tests, whereas continuous variables were compared using *t-*tests or nonparametric Mann–Whitney *U* tests. All statistical analyses were performed using IBM SPSS Statistics, version 26.0 (IBM Corp., Armonk, NY, USA), and were considered significant at *p* < 0.05 (two-tailed).

Propensity score (PS) matching [22, 23] was used to obtain balance between the corticosteroid (case) and non-corticosteroid (control) groups. In the total population, we used 1:1 nearest-neighbor matching without replacement within a match tolerance width of 0.001; in patients with IMV and/or shock and in patients without IMV nor shock, the match tolerance width was set at 0.005. Variables were chosen for inclusion in the PS calculation according to the methods of Brookhart et al. [24], and we included those associated with the case group and outcome (Center, age, sex, diabetes mellitus, ischemic heart disease, hypertension, COPD, cancer, SOFA score, septic shock, and initial appropriate treatment). In the total population, adequate model fit with discrimination and calibration of the PS was demonstrated by logistic modeling including covariates (goodness-of-fit, *p* = 0.792) and also in patients with IMV and/or shock (goodness-of-fit, *p* = 0.614) and in patients without IMV nor shock (goodness-of-fit, *p* = 0.715). We performed three subgroup exploratory analyses for patients with septic shock only, IMV requirement only, and both septic shock and IMV requirement (see Additional file 1: Content for further details). Multiple imputation [25] was used for missing covariates in PS matching.

Survival curves for patients with and without corticosteroids were obtained by the Kaplan–Meier method and compared using the Gehan–Breslow–Wilcoxon test. We used Cox proportional hazard regression models [26] for the 28-day mortality. Hazard ratios (HRs) and 95% confidence intervals (95% CIs) were calculated.

## Results

**Fig. 1:**
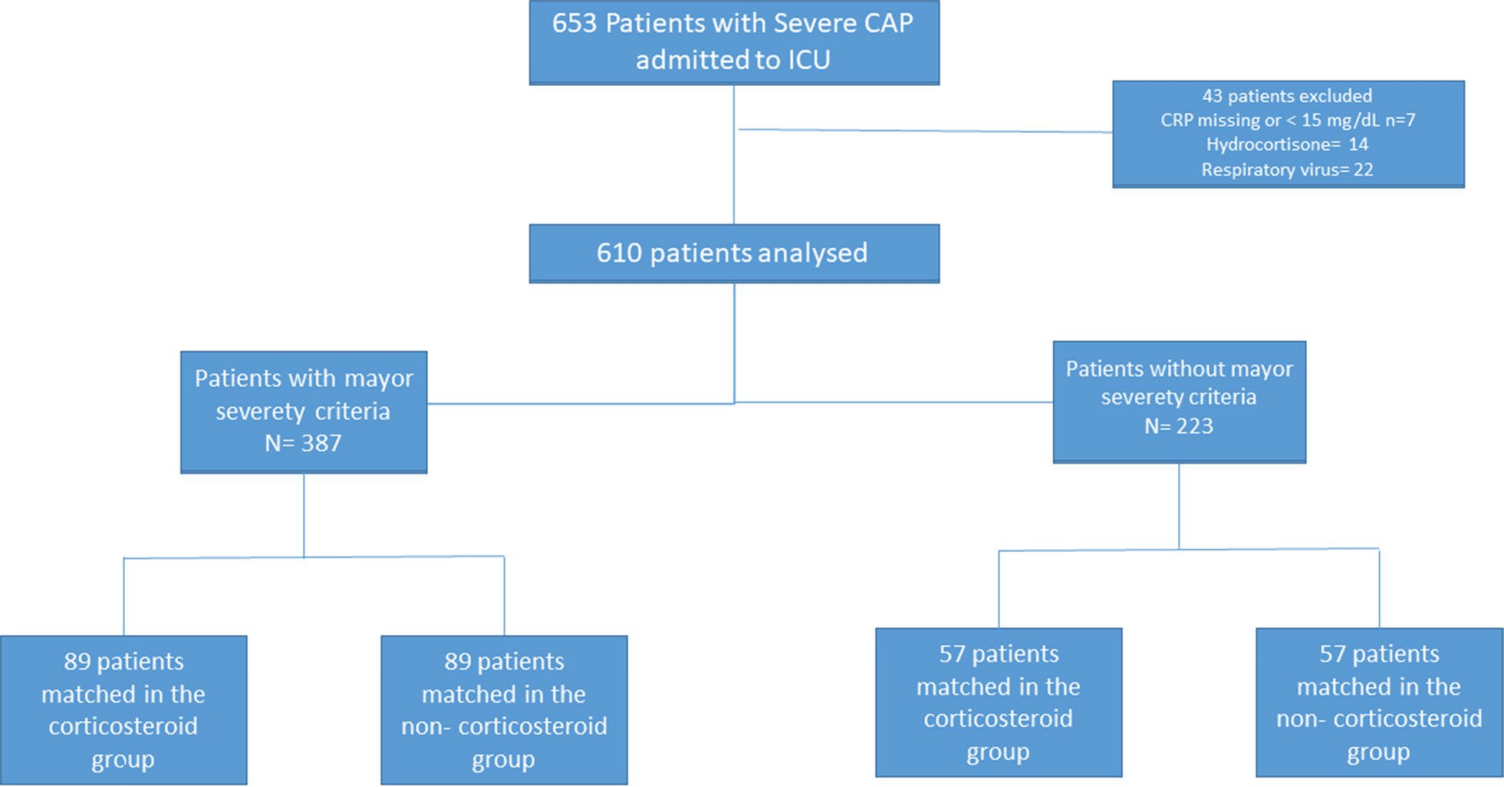
Flowchart. CAP, community-acquired pneumonia; CRP: C-reactive protein; ICU, intensive care unit; IMV, invasive mechanical ventilation; PS, propensity score

Of the 610 included patients, 198 (32%) received corticosteroids (Fig. 1) and 387 had major criteria for severe CAP. Microbial isolation was reported in 227 patients (55%) who did not receive corticosteroids and in 99 (50%) who received corticosteroids. The most common microorganism was *Streptococcus pneumoniae*, followed by *Staphylococcus aureus*, *Enterobacteriaceae*, and *Haemophilus influenzae*. For the entire population, before PS matching, antibiotic treatment was appropriate for 302 patients (92%) in the non-corticosteroid group and 115 patients (86%) in the corticosteroid group (eTable 1).

The main baseline characteristics of patients with major criteria for severe CAP are described in Table 1. Patients who received corticosteroids were more commonly male, more often had comorbidities (e.g., diabetes mellitus, cancer or chronic obstructive pulmonary disease), presented significantly higher PSI and SOFA scores, and lower PaO_2_/FiO_2_, heart rate, and creatinine levels. Antibiotic treatment was appropriate for 302 patients (92%) in the non-corticosteroid group and 115 patients (86%) in the corticosteroid group (Table 1). No significant differences were found in all outcome variables.

**Table 1:** Patient characteristics in the full cohort of patients with septic shock and/or IMV requirements and in the propensity score matching sample

Variable	Before PS matching (*N* = 387			After PS matching (*N* = 178)		
	Corticosteroids			Corticosteroids		
	No	Yes		No	Yes	
	*N* = 268	*N* = 119	*p* value	*N* = 89	*N* = 89	*p* value
Age (years), median (Q1; Q3)	68.5 (54.5; 80)	72 (59; 81)	0.126	73 (56; 84)	72 (59; 83)	0.839
Male sex, *n* (%)	115 (43)	68 (57)	**0.010**	50 (56)	48 (54)	0.763
Current smoking habit, *n* (%)	86 (33)	34 (30)	0.489	27 (32)	24 (28)	0.616
Current alcohol abuse, *n* (%)	37 (21)	8 (13)	0.190	10 (21)	6 (13)	0.273
Comorbidities, *n* (%)						
Diabetes mellitus	63 (24)	17 (14)	**0.033**	15 (17)	15 (17)	1.000
Ischemic heart disease	31 (18)	21 (18)	0.880	13 (15)	13 (15)	1.000
Hypertension	64 (38)	56 (47)	0.110	36 (40)	37 (42)	0.879
COPD	58 (22)	41 (35)	**0.012**	27 (30)	27 (30)	1.000
Cancer	32 (12)	25 (21)	**0.028**	11 (12)	13 (15)	0.661
SOFA score, median (Q1; Q3)	5 (3; 6)	5 (4; 7)	**0.003**	5 (4; 7)	5 (3; 7)	0.483
Pneumonia Severity Index, median (Q1; Q3)	127.5 (107; 154.5)	140 (119; 163)	**0.007**	140 (121; 157)	135 (116; 159)	0.500
PaO_2_/FiO_2_ < 250, *n* (%)	110 (42)	70 (59)	**0.003**	51 (44)	51 (57)	0.415
Altered mental status, *n* (%)	83 (31)	31 (26)	0.296	39 (44)	23 (26)	**0.010**
Respiratory rate, median (Q1; Q3)	28 (24; 32)	26 (21; 30)	0.058	28 (20; 35)	25 (20; 30)	0.276
Heart rate, median (Q1; Q3)	110 (28; 95)	100 (81; 114)	**0.004**	107 (90; 130)	98 (85; 111)	**0.034**
Temperature (°C), median (Q1; Q3)	37 (36.2; 38)	37.4 (36.7; 38)	0.161	37 (36.3; 38)	37.4 (36.8; 38)	0.092
Creatinine (mg/dL), median (Q1; Q3)	1.4 (1; 1.9)	1.2 (0.8; 1.6)	**0.001**	1.3 (1; 1.9)	1.1 (0.8; 1.6)	**0.032**
CRP (mg/dl), median (Q1; Q3)	29 (23.1; 37.1)	30.3 (24.4; 44.1)	0.274	29.4 (24.8; 37.3)	30 (24.3; 43.9)	0.961
White blood cell count (10^9^ cells/L), median (Q1; Q3)	12.6 (7.6; 19)	14.6 (7.9; 20.9)	0.259	12.8 (6.4; 17.9)	16 (9.2; 20.7)	0.131
Need of IMV, *n* (%)	134 (52)	50 (42)	0.073	42 (50)	32 (36)	0.062
Septic shock, *n* (%)	197 (74)	107 (90)	**0.001**	74 (83)	79 (89)	0.281
Polymicrobial infection, *n* (%)	8 (3)	10 (8)	**0.020**	4 (4)	8 (9)	0.232
Initial appropriate treatment, *n* (%)	237 (92)	97 (88)	0.319	78 (88)	85 (96)	0.059

Boldface entries indicate statistical significance

CAP, community-acquired pneumonia; COPD, chronic obstructive pulmonary disease; CRP: C-reactive protein; DM, Diabetes mellitus; IHD, ischemic heart disease; IMV, invasive mechanical ventilation; PS, propensity score; Q1, first quartile; Q3, third quartile; SOFA: Sequential Organ Failure Assessment. Percentages calculated with non-missing data only. *p* values marked in bold indicate numbers that are statistically significant on the 95% confidence limit

**Fig. 2:**
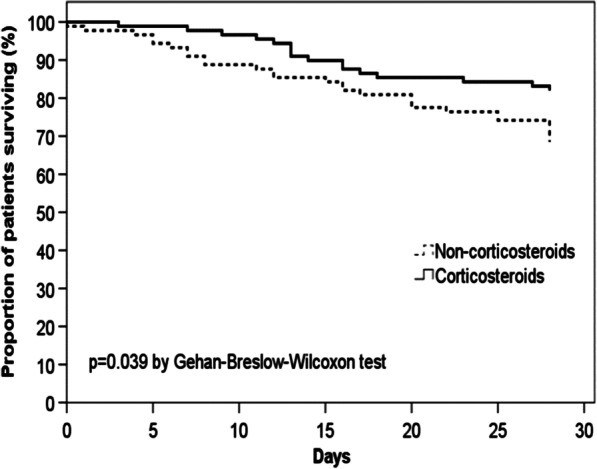
Kaplan–Meier survival curve after PS matching

PS matching was performed in patients with major criteria for severe CAP, resulting in 89 patients per group with significant differences in mental status and creatinine level alterations. The 28-day mortality was lower among patients who received corticosteroids (16 patients [18%]) than among those who did not (28 patients [31%]; *p* < 0.05). Cox regression analyses revealed that corticosteroids reduced the 28-day mortality risk, giving an HR of 0.53 (95% CI 0.29–0.98). The Kaplan–Meier survival curves for patients receiving IMV and/or experiencing shock are shown in Fig. 2.

For patients who did not meet major criteria for severe CAP, PS matching resulted in 57 patients per group. Twenty-eight-day mortality was similar between groups (16% [*n* = 9] in the corticosteroid group vs 18% [*n* = 10] in the non-corticosteroid group, HR 0.88 (95% CI 0.32–2.36).

Sensitivity analysis was performed in all patients admitted to the ICU and did not find significant differences between groups (etable 2).

Among patients who received corticosteroids, hospital stay was shorter only in those who met major severity criteria (Table 2).

**Table 2:** Outcomes in the full cohort of patients with septic shock and/or IMV requirements and in the propensity score matching sample

	Before PS matching (*N* = 387)			After PS matching (*N* = 178)		
	Corticosteroids			Corticosteroids		
	No	Yes		No	Yes	
	*N* = 268	*N* = 119	*p* value	*N* = 89	*N* = 89	*p* value
28-day mortality, *n* (%)	65 (24)	24 (20)	0.368	28 (31)	16 (18)	**0.037**
Hospital length of stay (days), median (Q1; Q3)	17 (10; 28)	14 (10; 25)	0.216	17 (10.5; 29)	13 (9; 20)	**0.027**

IMV, invasive mechanical ventilation; PS, propensity score; Q1, first quartile; Q3, third quartile. Percentages calculated with non-missing data only. *p* values marked in bold indicate numbers that are statistically significant on the 95% confidence limit

## Discussion

In the present study, mortality benefits were only observed among the most severely ill patients with CAP receiving corticosteroid therapy. The 28-day mortality did not differ with corticosteroids use in either the matched or unmatched analysis for patients in ICU with a high inflammatory response; however, when we analyzed only patients who required IMV and/or presented septic shock, mortality was significantly lower among those who received corticosteroids. These data from a multinational and real-world setting support the results of a prior meta-analysis.

Corticosteroid treatment for CAP is a controversial topic. Despite several studies showing improved outcomes, such as less treatment failure, shorter hospital stays, shorter time to clinical stability [19, 27], or reduced risk of cardiovascular events [28], only one has shown improved mortality [29]. This may be because many studies have lacked the statistical power to find significance differences. Improved mortality has been observed in several meta-analyses, mainly for severe CAP [15, 16, 30–32], but concerns about non-reproducible results and differences in baseline characteristics have been raised for the two studies [29, 33] with the greatest positive results that drove the conclusions. A major limitation of these studies and meta-analyses is the fact that different criteria were used for severe pneumonia (i.e., admission to ICU, ATS/IDSA criteria, septic shock, invasive mechanical ventilation requirement, or high severity scores). These differences have led to contradictory suggestions in clinical practice guidelines. While guidelines for the diagnosis and management of critical illness-related corticosteroid insufficiency (CIRCI) by the Society of Critical Care Medicine and European Society of Intensive Care Medicine favor corticosteroid use in CAP [34], ATS/IDSA guidance for CAP management continues to advise against corticosteroid use [4].

We took care to select only those patients who could benefit from corticosteroids. Corticosteroids are thought to immunomodulate the disproportionate inflammatory response, so we only included patients with a measurably elevated inflammatory response (CRP > 15 mg/dL) [19, 35]. We excluded patients with viral pneumonia because several analyses have shown that corticosteroids may increase mortality in patients admitted for severe influenza [36]; however, this is somewhat controversial because dexamethasone has been shown to reduce mortality in patients with COVID-19 [37, 38]. Patients who received hydrocortisone were excluded as well, based on the results of a meta-analysis showing that patients who received hydrocortisone did not present benefits in terms of mortality [39].

We found reduced mortality associated with corticosteroid use in only the patients considered most severely ill according to the ATS/IDSA criteria. Corticosteroids have been evaluated in other related severe diseases, such as acute respiratory distress syndrome (ARDS) [40] or septic shock, with those results also open to debate. In the last Surviving Sepsis campaign, it was stated that corticosteroids were indicated in cases of septic shock when hemodynamic stability was not achieved despite adequate fluid resuscitation and vasopressor support [41]. Recent positive results when using dexamethasone in ARDS have re-opened the debate about corticosteroid use in this setting [40]. We did not find any difference in 28-day mortality in specific sub-populations (septic shock or IMV requirements alone) when analyzed separately, but we could only match small numbers of patients in each group, which makes it difficult to reach a firm conclusion. Further studies are needed to evaluate if corticosteroids reduce the risk of death in CAP by reducing pulmonary inflammation or improving hemodynamic parameters.

A major strength of the present research is that we could reproduce, in a real-world, multinational setting, results that have previously only been shown in meta-analyses. However, our study still had several limitations. First, the observational design means that patients may have received corticosteroids for a reason other than severe CAP. Although we tried to recreate the conditions of a clinical trial as far as possible, corticosteroid treatments were not protocoled, and so patients who received corticosteroids were not homogeneous in terms of time until first corticosteroid dose, total dose, and type of corticosteroids. Second, despite exhaustive PS matching for underlying conditions, severity criteria, treatment adequacy, center, etc., some differences remained between the groups. Finally, we established time windows to include or exclude patients. These time windows can be found in previous clinical trials; however, in our case they may have led us to select certain patients and not others. Nevertheless, these limitations also reflect the real-life scenario. Our results must be considered with care, given the controversial results in clinical trials (including one unpublished trial, NCT01283009). However, they may help to develop new clinical trials including only the most severely ill populations with high inflammatory responses.

## Conclusions

The mortality benefits of corticosteroid treatment for CAP, as previously reported in a meta-analysis, only appear to be observed in the most severely ill patients who have a high inflammatory response in real-world settings. In the absence of patients with severe CAP having an IMV requirement or developing septic shock, corticosteroids are not associated with lower mortality.

## Supplementary Information

**Additional file 1.** Supplementary Online Content with additional methods, baseline charachteristics and outcomes is available.

## Data Availability

The datasets used and/or analyzed during the current study are available from the corresponding author on reasonable request.

## Abbreviations

ATS/IDSA: American Thoracic Society and Infectious Diseases Society of America
CAP: Community-acquired pneumonia
CIRCI: Critical illness-related corticosteroid insufficiency
CRP: C-reactive protein
ICU: Intensive care unit
IMV: Invasive mechanical ventilation
PSI: Pneumonia Severity Index
PS: Propensity score
SOFA: Sepsis-related Organ Failure Assessment
