# Supplementary material for: Real-world corticosteroid use in severe pneumonia: a propensity-score-matched study
Source: Crit Care. 2021 Dec 16;25:432. doi: 10.1186/s13054-021-03840-x (PMC8674860; doi:10.1186/s13054-021-03840-x)
Supplement: Supplementary file 1 — Additional file 1. Supplementary Online Content with additional methods, baseline charachteristics and outcomes is available. [file 13054_2021_3840_MOESM1_ESM.docx]

**Supplementary** **Online Content**

**Real-world corticosteroid use in severe pneumonia: a propensity score matched study**

**METHODS**

**Microbiologic tests**

The following microbiological tests were carried out: urinary antigens for *Legionella pneumophila*and *Streptococcus pneumoniae*, sputum Gram stain (if < 10 epithelial cells and > 25 leukocytes per field; magnification ×100), and culture. We also performed paired blood cultures and paired serological studies for *Chlamydophila pneumoniae*, *Mycoplasma pneumoniae*, *Coxiella burnetti*, and *Legionella pneumophila*. Nasopharyngeal swabs were taken to detect viral nucleic acids, based on clinical suspicion. Invasive samples were taken if requested by the attending physician.

**Statistical analysis**

In addition to the primary analyses, we also performed three subgroup exploratory analyses for patients with only septic shock, patients with only invasive mechanical ventilation (IMV) requirement, and patients with septic shock and IMV requirement. We used 1:1 nearest-neighbor propensity score (PS) ^1,2^ matching without replacement within a match tolerance width of 0.005. In the subgroups with septic shock only, IMV requirement only, and both septic shock and IMV requirement, adequate model fit was demonstrated by the corresponding logistic model, including covariates, which yielded goodness-of-fit values of p = 0.988, p = 0.760, and p = 0.231, respectively.

1. Austin PC. An Introduction to Propensity Score Methods for Reducing the Effects of Confounding in Observational Studies. *Multivar Behav Res*. 2011;46(3):399-424. doi:10.1080/00273171.2011.568786

2. Rosenbaum PR, Rubin DB. The Central Role of the Propensity Score in Observational Studies for Causal Effects. *Biometrika*. 1983;70(1):41-55. doi:10.2307/2335942

**Table 1. Patient characteristics in the overall population**

| **Variable** | **Overall population** | | | |
| --- | --- | --- | --- | --- |
|  | **Before PS Matching** | | **After PS Matching** | |
|  | **Corticosteroids** | | **Corticosteroids** | |
|  | **No** | **Yes** | **No** | **Yes** |
|  | N=412 | N=198 | N=117 | N=117 |
| Age (years), median (Q1; Q3) | 72 (57; 82) | 73 (61; 83) | 74 (58; 83) | 72 (59; 83) |
| Male sex, n (%) | 172 (42)** | 120 (61)** | 62 (53) | 63 (54) |
| Current smoking habit, n (%) | 120 (30) | 63 (34) | 25 (23) | 36 (33) |
| Current alcohol abuse, n (%) | 48 (18) | 15 (16) | 7 (13) | 8 (14) |
| Comorbidities, n (%) |  |  |  |  |
| Diabetes mellitus | 103 (25) | 38 (19) | 23 (20) | 25 (21) |
| Ischemic heart disease | 46 (17) | 30 (15) | 23 (20) | 18 (15) |
| Hypertension | 105 (40) | 92 (46) | 45 (38) | 46 (39) |
| COPD | 96 (24)* | 70 (35)* | 30 (26) | 38 (32) |
| Cancer | 50 (12)* | 40 (20)* | 16 (14) | 22 (19) |
| SOFA score, , median (Q1; Q3) | 4 (3; 6)* | 5 (3; 7)* | 4 (2.6; 6) | 4 (3; 5) |
| Pneumonia severity Index , median (Q1; Q3) | 130 (111; 152)* | 138(116; 159)* | 135.5 (114; 155) | 135 (106; 155) |
| Altered mental status, n (%) | 133 (33) | 52 (26) | 36 (31) | 30 (26) |
| Respiratory rate, median (Q1; Q3) | 30 (24; 36)** | 26 (21; 29)** | 28 (24; 36)* | 25 (21; 28)* |
| Temperature (˚C), median (Q1; Q3) | 37.3 (36.3; 38.1) | 37.4 (36.8; 38) | 37.3 (36.2; 38) | 37.4 (37; 38) |
| Creatinine (mg/dL), median (Q1; Q3) | 1.3 (0.9; 1.9)** | 1.1 (0.8; 1.6)** | 1.1 (0.8; 1.8)* | 1. 0 (0.8; 1.4)* |
| CRP (mg/dl), median (Q1; Q3) | 28.1 (22.6; 36.5) | 29 (23.9; 39.6) | 28.3 (24.8; 36) | 28.2 (24; 39) |
| White blood cell count (10^9^ cells/L), median (Q1; Q3) | 12.7 (7.8; 18.4) | 14.5 (8.8; 20.1) | 13.9 (7.9; 19.9) | 14.9 (8.8; 19.6) |
| Need of IMV, n (%) | 134 (34)* | 50 (25)* | 29 (26) | 20 (17) |
| Septic shock, n (%) | 197 (48) | 107 (54) | 60 (51) | 56 (48) |
| Polymicrobial infection, n (%) | 13 (3)* | 15 (8)* | 5 (4) | 8 (7) |
| Initial appropriate treatment, n (%) | 302 (92) | 115 (86) | 104 (89) | 112 (96) |

Abbreviations: CAP = community-acquired pneumonia; COPD = chronic obstructive pulmonary disease; CRP: C-reactive protein; DM = Diabetes mellitus; IHD = ischemic heart disease; IMV = invasive mechanical ventilation; PS = propensity score; Q1 = first quartile; Q3 = third quartile; SOFA: Sequential Organ Failure Assessment. Percentages calculated with non-missing data only. * p value for comparison between corticosteroids and non-corticosteroids groups <0.05. ** p value for comparison between corticosteroids and non-corticosteroids groups <0.001.

**eTable 2. Outcomes in overall population and in patients who required invasive mechanical ventilation and/or had septic shock**

| **Variable** |  |  | **Before PS Matching** | | | **After PS Matching** | |
| --- | --- | --- | --- | --- | --- | --- | --- |
|  |  |  | **Corticosteroids** | | | **Corticosteroids** | |
|  | **Group** | **Statistic** | **No** | **Yes** | **No** | | **Yes** |
| 28-day mortality |  |  |  |  |  | |  |
|  | Overall population | n/N (%) | 84/410 (20) | 38/198 (19) | 21/116 (18) | | 20/117 (17) |
|  | Patients with septic shock and/or IMV requirement | n/N (%) | 65/267 (24) | 24/119 (20) | 28/89 (31)* | | 16/89 (18)* |
|  | Patients with only septic shock | n/N (%) | 23/124 (19) | 13/69 (19) | 8/45 (18) | | 7/45 (16) |
|  | Patients with only IMV requirement | n/N (%) | 16/68 (24) | 2/12 (17) | 1/4 (25) | | 0/4 (0) |
|  | Patients with septic shock and IMV requirement | n/N (%) | 23/63 (37) | 9/38 (24) | 9/17 (53) | | 5/17 (30) |
| Hospital length of stay (days) |  |  |  |  |  | |  |
|  | Overall population | N | 324 | 155 | 115 | | 113 |
|  |  | Median (Q1; Q3) | 15 (10; 26) | 13 (9; 22) | 15 (9; 21) | | 12 (8; 19) |
|  | Patients with septic shock and/or IMV requirement | N | 265 | 114 | 88 | | 84 |
|  |  | Median (Q1; Q3) | 17 (10; 28) | 14 (10; 25) | 17 (10.5; 29)* | | 13 (9; 20)* |

Abbreviations: IMV = invasive mechanical ventilation; PS = propensity score; Q1 = first quartile; Q3 = third quartile. 28-day mortality calculated as the ratio between the total number of patient deaths (n) in each group and the total number of patients (N) in each group multiplied by 100. For some patients, the data (septic shock, IMV requirement, 28-day mortality, and/or hospital length of stay) were not available. * p value for comparison between corticosteroids and non-corticosteroids groups <0.05.
